# Smoking cessation advice from healthcare professionals helps those in the contemplation and preparation stage: An application with transtheoretical model underpinning in a community-based program

**DOI:** 10.18332/tid/123427

**Published:** 2020-07-02

**Authors:** Pallop Siewchaisakul, Dih-Ling Luh, Sherry Y. H. Chiu, Amy M. F. Yen, Chih-Dao Chen, Hsiu-Hsi Chen

**Affiliations:** 1 School of Dentistry, College of Oral Medicine, Taipei Medical University, Taipei, Taiwan; 2 School of Oral Hygiene, College of Oral Medicine, Taipei Medical University, Taipei, Taiwan; 3 Oral Health Care Research Center, College of Oral Medicine, Taipei Medical University, Taipei, Taiwan; 4 Department of Public Health, Chung Shan Medical University, Taichung, Taiwan; 5 Department of Family and Community Medicine, Chung Shan Medical University Hospital, Taichung, Taiwan; 6 Department of Health Care Management, College of Management, Chang Gung University, Taoyuan City, Taiwan; 7 Department of Internal Medicine, Kaohsiung Chang Gung Memorial Hospital, Kaohsiung City, Taiwan; 8 Department of Family Medicine, Far Eastern Memorial Hospital, Taipei, Taiwan; 9 Institute of Epidemiology and Preventive Medicine, College of Public Health, National Taiwan University, Taipei, Taiwan

**Keywords:** Markov model, smoking cessation, transtheoretical model, physician, nurse

## Abstract

**INTRODUCTION:**

The efficacy of smoking cessation intervention has been proven with randomized controlled trials. Our study aims to elucidate the effects of the delivery method of smoking cessation advice on the process of stage of changes with transtheorectical model underpinning in a community setting.

**METHODS:**

A total of 436 subjects were recruited in a quasi-experimental untreated control design study, with 46 receiving advice from healthcare professionals (HCP group) and 390 in the control group, in 2003, Nantou, Taiwan. A discrete time Markov model was used to quantify the multi-state process of smoking cessation in light of the transtheorectical model. Multiple polytomous logistic regression models were simultaneously applied to different transitions.

**RESULTS:**

The estimated forward transition probabilities were higher in the HCP group compared to their counterparts in the control group. On the other hand, the backward transition probabilities were smaller in the HCP group. After adjusting for confounding factors, HCP had a 4.3-fold (95% CI: 2.21–8.46) odds ratio of moving forward from the contemplation stage, and 2.4-fold odds ratio (95% CI: 1.03–4.42) from the preparation stage. Elderly people were more reluctant to change from precontemplation (AOR=0.50; 95% CI: 0.34–0.74) and contemplation (AOR=0.58; 95% CI: 0.44–0.84), but once in the preparation stage, they were more likely to take action (AOR=1.28; 95% CI: 1.01–1.83). For those in the preparation stage, longer smoking years had a negative effect on taking action (AOR=0.74; 95% CI: 0.52–0.99), but cessation advice from others enhanced the likelihood to take action (AOR=1.36; 95% CI: 1.01–1.99).

**CONCLUSIONS:**

The direct advice on smoking cessation from healthcare professionals enforced the net forward transition towards smoking cessation, especially the transition from contemplation and preparation. The proposed Markov regression model assessed the net effect of different intervention approaches allowing for the simultaneous consideration of multiple transitions and the effects of other confounders.

## INTRODUCTION

Smoking has been recognized as the second leading risk factor for global disease burden, accounting for 7.1 million deaths and 182 million disability-adjusted life-years in 2017, after high systolic blood pressure^1^. Those who begin smoking young and continue smoking could reduce life expectancy by almost 10 years^2,3^. Tobacco control efforts and smoking cessation promotion have been advocated worldwide^4^. In addition to population-level approaches, such as legislation and tobacco taxation, the individual-level approaches were promoted with pharmaceutical and non-pharmaceutical means to help people intending to quit smoking. The efficacy of the cessation advice given to those intending to quit smoking from healthcare professionals, including physicians, pharmacists, nurses, and dentists, has been demonstrated^5-8^. It is of paramount importance to evaluate whether healthcare professionals also help smokers to quit in the general population.

Quitting smoking is a behavior change that makes progress through a series of stages. The transtheoretical model (TTM) has been proposed to describe the process for the behavior of quitting smoking since 1980s^9-14^. Understanding how different intervention programs affect the process of behavior change facilitates planning of interventions. The efficacy of the delivery method of smoking cessation advice is of interest in order to design an efficient intervention program for smoking cessation. However, not only the endpoint of successful smoking cessation is of interest, but also the process of behavior change is important. Up to now, most studies assessed the intervention in terms of whether it can increase the probability of successful smoking cessation, rather than how the intervention affects the process of smoking cessation.

Research has been conducted to investigate the dynamics associated with the progressive direction from earlier stages, like ‘precontemplation’ or ‘contemplation’, to ‘preparation’, and to ‘action to smoking cessation’, or factors for the deterioration from ‘preparation’ back to ‘contemplation’, or even to ‘precontemplation’. In addition to the process of smoking cessation, the unique cyclic property of the TTM focusing on not only from precontemplation to action but also on changes in the reverse direction, describes well the hesitation and action to quit smoking^15-17^. It would be interesting to simultaneously elucidate the stage-specific effects of these factors. By doing so, it can help to design a stage-specific smoking cessation intervention program.

In this study, we aimed to assess the effect of smoking cessation advice given by healthcare professionals on the process of stage of changes with TTM underpinning in a community setting with a quasi-experimental untreated control design with pre-test and post-test. We used a discrete time four-state Markov Chain model to estimate the transition probabilities between stages allowing for forward and backward processes with adjustment of smoking related confounding factors.

## METHODS

### Study subjects

Subjects were selected from those who attended the community-based integrated screening (CIS) program in Nantou County, which is located in central Taiwan. Geographically, Nantou County is surrounded by mountains, the only county without train connection. The geographical characteristics and inconvenient transportation situation have resulted in a lower level of medical resources. The CIS program was firstly initiated in Keelung since 1999^18,19^. It was an outreach program and usually conducted in the resident activity center in the specific area (village/town/district). The report of examination was given in person with a team of health professionals (including physicians and public health nurses) in the same site 1–2 weeks after the screening date. The Health Bureau of Nantou County launched its CIS program in 2001, after the Taiwan 921 Jiji earthquake heavily damaged this area and killed thousands of residents in 1999. Information on lifestyle (such as smoking, alcohol drinking, exercise and dietary habits) and personal and family history was obtained in a structured questionnaire administered by well-trained public health nurses or social volunteers who participated in the CIS program.

Varying health promotion programs were embedded in the Nantou CIS program^20,21^. The current study was initiated by the Nantou Health Bureau, which called for a proposal to evaluate whether the intervention provided by healthcare professionals (HCP) would enhance attitudes towards smoking cessation in 2003. All participants gave written inform consent to the Nantou Health Bureau to use the data for healthcare management. Data of personal identification were removed or de-identified by the staff of the health bureau before being released. This study was approved by Nantou Health Bureau to meet any ethical requirement mandated by Taiwanese government.

### Intervention

**Figure 1:**
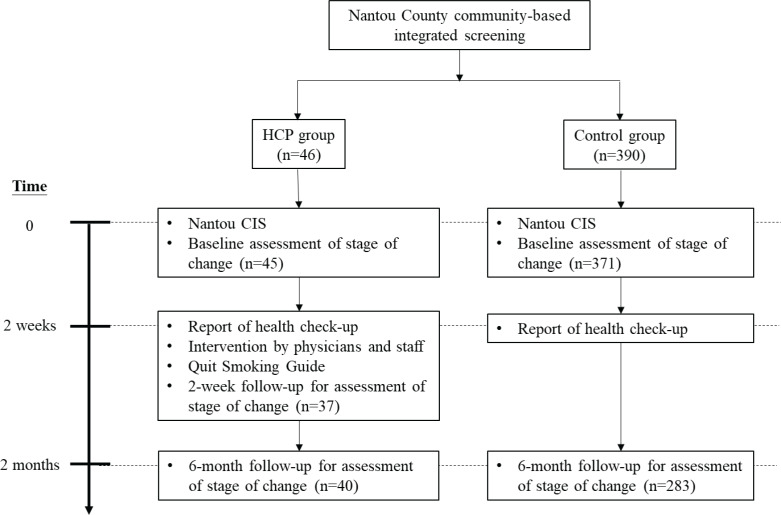
Participant flow for the quasi-experimental design study on smoking cessation advice, Nantou 2003

Among the 13 towns and villages in Nantou County, we selected Lugu Village to conduct the advice program given by healthcare professionals (HCP group), and other villages/towns that showed willingness to participate were selected as the control group. Because there were only 2.6% females among the smokers in this program, we included male smokers only in the current study. A total of 436 subjects were recruited in this quasi-experimental untreated control design study, including 46 in the HCP group and 390 in the control group. The study flowchart is shown in Figure 1.

Before the CIS program, the consensus meeting was held in Nantou Health Bureau chaired by one coauthor (LDL) who is specialized in health education and health promotion. The meeting invited physicians and public health nurses in Lugu Village. A course on smoking control, health education, and the application of transtheoretical models for behavior change was firstly taught. There was panel discussion between the lecturer and staff in the HCP for the design of an advice document on smoking cessation, ‘Quit Smoking Guide’, an assessment table for smoking cessation, and a survey for those willing to quit smoking.

‘Quit Smoking Guide’ together with a personal report of a health checkup was directly provided to subjects in the HCP when they returned in person to obtain their reports on the screening results. Physicians pointed out the hazards of smoking on health during consultation based on a participant’s personal examination report, especially when the participants had any abnormal findings or personal and family disease history that was related to smoking. Public health nurses reassessed their stage of change of smoking cessation. Participants in the control group, either obtained their reports in person or by post, and had no particular intervention for quitting smoking.

The stage of smoking cessation of each participant was assessed at baseline based on their responses to the smoking related questions in the CIS program (see below). Subjects in the HCP were reassessed when they obtained their screening report two weeks after the CIS (the first follow-up). Two months after the CIS program, another reassessment of stage of change of all participants in both HCP (the second follow-up) and control groups (the first follow-up) was done by a telephone survey using the same questionnaire. Finally, a total of 40 and 283 male smokers in the HCP and control groups, respectively, who responded to questions at both baseline and at the two months follow-up survey were included in the current analysis.

### Operational definition of stage with TTM underpinning

In the CIS framework, we routinely collected data on demographic variables, questionnaires, and results of biochemical examination of screening items. For the health promotion program for smoking cessation, some smoking related questions were asked to current smokers, including smoking commencement age, time of first cigarette in the morning, experience of smoking cessation advice from others in previous six months, the intention to quit smoking, and the time schedule for quitting (for those who gave a positive answer to the previous question).

Following the TTM^10,11^, the stage of smoking cessation, including ‘Precontemplation’, ‘Contemplation’, ‘Preparation’, and ‘Action’ was assigned to each participating smoker based on the responses to the associated questions in the questionnaire. The operation definition has been described previously^20,21^. Briefly, ‘Precontemplation’ was assigned to those who never considered quitting. ‘Contemplation’ was assigned to those who ever considered quitting and intended to do so in the following six months. ‘Preparation’ was assigned to those ever considered quitting and were about to quit in the coming month. ‘Action’ was assigned to those who responded that they had quit smoking.

### Statistical analysis

To compare the characteristics of participants, between the HCP and the control groups, we used Student’s t-test for continuous variables and chi-squared test for categorical variables. We applied a four-state Markov Chain model with state space of ‘Precontemplation’, ‘Contemplation’, ‘Preparation’, and ‘Action’, to estimate the probabilities of transitions between states with TTM underpinning^22^. In order to investigate the effects of the advice program and other smoking related factors, such as commencement age of smoking, duration of smoking, and time to first cigarette in the morning, on the transitions between states, we simultaneously applied polytomous logistic regression models in the Markov Chain model. The net odds ratios (ORs) of covariates considering the net force of forward and backward transitions from any state were reported^23^. Details of the four-state Markov model and its regression form with covariates are given in the Supplementary file. All statistical analyses were conducted in SAS version 9.4. An alpha level of 0.05 was set as statistically significant level.

## RESULTS

Table 1 shows the sociodemographic characteristics of the HCP and control groups. Subjects in the HCP group were older by 5 years than the control group (p<0.001). Because the duration of smoking was related to age, we also found 5 years longer smoking years in the HCP group (p=0.017). Participants in both groups started smoking at an age of 21–22 years (p=0.482). The proportion of first cigarette within 30 minutes of waking up in the morning was about 60% in both groups (p=0.746), while 62% and 64% of participants in the HCP and control groups, respectively, received smoking cessation advice from others (p=0.636).

**Table 1:** Sociodemographic characteristics of participants in the smoking cessation program, Nantou 2003 (N=323)

*Variables*	*HCP group (N=40)*		*Control group (N=283)*		*p*
**Age** (years), mean ±SD	71.00	±7.22	66.15	±12.22	<0.001
**Smoking commencement age** (years), mean ±SD	21.19	±19.09	21.98	±21.22	0.482
**Duration of smoking** (years), mean ±SD	47.73	±10.5	42.13	±±13.61	0.017
**Minutes to first cigarette in the morning,** n %					
<30	25	64.1	160	58.8	0.746
≥30	14	35.9	112	41.2	0.636
**Smoking cessation advice from others,** n %					
No	15	38.5	96	36.1	
Yes	24	61.5	170	63.9	

**Figure 2:**
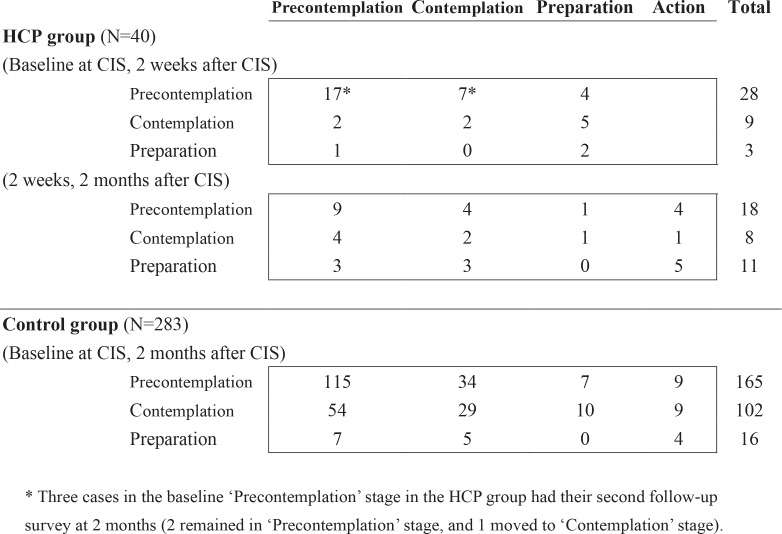
Stage changes before and after intervention for smoking cessation for male smokers by means of smoking cessation advice, Nantou 2003 (N=323)

Figure 2 shows the data layout of stage changes at baseline and follow-up survey. In the HCP group, 28 (70%), 9 (22.5%), and 3 (7.5%) men were initially in the stage of precontemplation, contemplation, and preparation, respectively. In the control group, there were initially 165 (58.3%), 102 (36.0%), and 16 (5.7%) men in the stage of precontemplation, contemplation, and preparation, respectively. The transitions after intervention by initial stages are shown in Figure 2.

Table 2 shows the estimated transition probabilities for the Nantou smoking cessation program by groups. It was estimated that around 27% (95% CI: 13–42%) of the men in the stage of precontemplation would change to contemplation, another 12% (95% CI: 3–23%) to preparation, and 11% (95% CI: 2–24%) every two weeks. Together with other forward probabilities from contemplation and preparation, we found that they were all higher than their counterparts in the control group. The regression probabilities in the HCP group were smaller than those in the control group.

**Table 2:** Estimated results of biweekly transition probability and the associated 95% credible interval (in parenthesis) between stage changes of smoking cessation, Nantou 2003 (N=323)

*Group*	*Cycle i+1*	*Stage of smoking cessation*			

	*Cycle i*	*Precontemplation*	*Contemplation*	*Preparation*	*Action*
**HCP**					
	Precontemplation	49.92% (---*)	26.77% (12.57, 41.8%)	12.17% (3.39, 22.73%)	11.14% (2.09, 24.31%)
	Contemplation	26.11% (3.21, 51.90%)	24.97% (---*)	42.45% (15.32, 73.23%)	6.47% (0.33, 19.31%)
	Preparation	33.88% (2.07, 70.26%)	16.18% (0.16, 43.56%)	29.78% (---*)	20.16% (0.84, 46.67%)
	Action	0%	0%	0%	100%
**Control**					
	Precontemplation	53.59% (---*)	35.64% (22.06, 52.23%)	7.22% (2.90, 12.07%)	3.55% (2.25, 4.97%)
	Contemplation	85.23% (68.05, 94.67%	9.35% (---*)	3.93% (0.25, 13.03%)	1.49% (0.22, 3.71%)
	Preparation	57.33% (0.33, 90.98%)	32.31% (0.27, 86.84%)	13.66% (---*)	5.7% (0.22, 18.42%)
	Action	0%	0%	0%	100%

* The complementary probability from each row. No confidence interval was calculated.

In the univariate analysis, the intervention group and all other confounding factors, including age, smoking commencement age, duration of smoking, first cigarette in the morning, and receiving cessation advice from others, significantly affected the net forward force from at least one stage (Supplementary Table S1). We therefore included these factors in the multi-variable model (Table 3). The results showed that smoking cessation advice provided by healthcare professionals had the higher net forward force from contemplation (AOR=4.29; 95% CI: 2.21–8.46) and preparation (AOR=2.40; 95% CI: 1.03–4.42). It is interesting to see that older age was negatively related to the net forward force from precontemplation (AOR=0.5; 95% CI: 0.34–0.74) and contemplation (AOR=0.58; 95% CI: 0.44–0.84), but once in the preparation stage, it was more likely to change toward the action stage (AOR=1.28; 95% CI: 1.01–1.83). For those in the preparation stage, longer smoking duration had a negative effect on taking action (AOR=0.74; 95% CI: 0.52–0.99), but subjects receiving cessation advice from others were more likely to take action (AOR=1.36; 95% CI: 1.01–1.99).

**Table 3:** Multivariate analysis of estimated odds ratio of transition departing from precontemplation, contemplation, and preparation, Nantou 2003 (N=323)

	*Precontemplation*		*Contemplation*		*Preparation*	

	*OR*	*95% CI*	*OR*	*95% CI*	*OR*	*95% CI*
**Intervention group HCP vs Control**	1.21	0.87–1.60	4.29*	2.21–8.46	2.40*	1.03–4.42
**Age per advanced year**	0.50*	0.34–0.74	0.58*	0.44–0.84	1.28*	1.01–1.83
**Smoking commencement age per advanced year**	0.90	0.75–1.07	1.19	0.80–1.68	1.73	0.96–3.49
**Duration of smoking per advanced year**	1.21	0.79–1.64	1.19	0.86-1.51	0.74*	0.52–0.99
**First cigarette in the morning** ≥**30 min vs <30 min**	1.34	0.71–2.18	0.44	0.14–1.12	1.04	0.47–2.17
**Cessation advice from others Yes vs No**	1.33	0.97–1.88	1.06	0.65–1.94	1.36*	1.01–1.99

* p<0.05.

## DISCUSSION

In this study, we assessed the effect of smoking cessation advice provided directly by healthcare professionals for male smokers who attended a community-based integrated screening program. We studied the effect of intervention on the dynamic stage of change with TTM underpinning. By using a four-state Markov regression model, we estimated the forward and backward transitions simultaneously, and quantified the net effect of the intervention program and covariates from different departing stages.

Our results showed that, without any particular intervention, around 47% of subjects in the precontemplation stage would depart, but there were also 85% of subjects in the contemplation and 57% in the preparation stage that would regress back to precontemplation in a two-week period. Same results were observed for subjects in contemplation and preparation. The forward and backward movements support the concept of a spiral model of change proposed by Prochaska et al.^24^.

The smoking cessation aids provided by healthcare professionals have been demonstrated as effective for those intending to stop smoking. Some studies on 3-day to 8-day residential programs with professional counseling and pharmacotherapy have demonstrated their effectiveness^25-29^. These studies targeted smokers who were willing to quit smoking or even in the state of nicotine dependence. Our study included a general population who participated in a community-based health check-up program. We demonstrated that advice from physicians and nurses enhanced the force towards smoking cessation, but only for those already in the contemplation and preparation stage and not significantly for those in the precontemplation stage.

Our results show that age, an earlier experience of cessation advice from others, and smoking years also affect the process towards smoking cessation. Age influenced differently according to the departing stage. We found older age was negatively associated with net forward force in precontemplation and contemplation, but a person was more likely to take action if in the preparation stage. This implies that for elderly people, the difficulty to access the resource of smoking cessation hindered them from thinking of quitting smoking. It would be more efficient to actively provide the tool of smoking cessation when they were prepared. In the meantime, those ever having cessation advice from others and with shorter smoking years were associated with higher forward transition for those in the preparation stage. These results are consistent with Ho et al.^25^ who provided residential treatment to those intending to stop smoking (namely, already beyond the contemplation stage) and found that the elderly with a longer smoking duration were more likely to quit^25^.

Carbonari et al.^15^ had applied the Markov chain analysis to modeling transitions in the process of smoking cessation in terms of TTM. They demonstrated that the first-order Markov model behaved better than the independence model, but the second-order model, which is more parameter demanding, did not improve the model fitting. Their results showed that people had a higher probability of progressing toward successfully maintained cessation than regressing or staying in the same stage, which also supports the spiral movement through the stages of changes. Our study further investigated the effects of covariates on these transitions by using first-order Markov regression models. Not only does one understand the net movement among smokers, but also the net benefit of intervention methods and personal smoking characteristics can be elucidated.

Professional smoking cessation interventions have become more and more popular. The established smoking cessation clinics have grown exponentially since 2005 in China, and 60% are in tertiary hospitals^30^. Some new models, such as decision support tools embedded in the e-information system, were introduced to facilitate the smoking cessation interventions^31^. These settings could benefit those who are willing to quit. From the public health point of view, targeting those in the general population not yet in the preparation stage could have a further impact in society. Our study showed that healthcare professionals could encourage smokers in the contemplation stage to step further. Clinicians and nurses in the primary prevention setting could work more efficiently when focusing on smokers who are in the contemplation stage. However, efforts from other fields were needed to encourage those prior to contemplation for quitting smoking.

### Limitations

Our study has some limitations. First, the stage of smoking cessation was observed at two and three time points for the control and HCP groups, respectively, in a 2-month interval. No relapse for smoking was observed. Therefore, in our study, we have to define the stage of action as the first time to quit smoking. Failure to maintain quitting smoking and recurrence of smoking were not considered in the current study. Second, this study did not include maintenance stage, which is usually treated as a successful endpoint of smoking cessation. Finally, we only included male smokers as the study population. The effect of advice from healthcare professionals on female smokers in the community could not be generalized from our study.

## CONCLUSIONS

Our results show that direct advice on smoking cessation from healthcare professionals enforced the net forward transition towards smoking cessation for those in the contemplation and preparation stage. The current study applied a four-state Markov regression model to assess the effect of different intervention approaches allowing for the simultaneous consideration of multiple transitions and the effects of other confounders.
