# Supplementary material for: Smoking cessation advice from healthcare professionals helps those in the contemplation and preparation stage: An application with transtheoretical model underpinning in a community-based program
Source: Tob Induc Dis. 2020 Jul 2;18:57. doi: 10.18332/tid/123427 (PMC7336865; doi:10.18332/tid/123427)
Supplement: Supplementary file 1 [file TID-18-57-s1.pdf]

Supplementary Materials

Four-state Markov Chain Model with transtheoretical model underpinning

We modeled the stage of changes of smoking cessation with a discrete time four-state Markov Chain model (Supplementary Figure 1) by defining state 1 as “Precontemplation”, state 2 as “Contemplation”, stage 3 as “Preparation”, and state 4 as “Action” [1]. The forward and backward transitions among four states were allowed before entering into “Action” state. In this study, “Action” was defined as the first event of quitting smoking. Therefore, once entering into “Action”, subjects stayed in “Action” without possibility of relapse to smoking. Two-week was used to be the length of one-step for the discrete-time Markov model. The one-step transition probability was expressed in the matrix form,

$$\mathbf{P} = \begin{matrix} 1 \\ 2 \\ 3 \\ 4 \end{matrix} \begin{pmatrix} P_{11} = (1 - P_{12} - P_{13} - P_{14}) & P_{12} & P_{13} & P_{14} \\ P_{21} & P_{22} = (1 - P_{22} - P_{23}) & P_{23} & P_{24} \\ P_{31} & P_{32} & P_{33} = (1 - P_{32} - P_{34}) & P_{34} \\ 0 & 0 & 0 & 1 \end{pmatrix}$$

P_{ij} represented the transition probability from state i to state j in one step. The summations of elements in each row were unity. Owing to the Markov property, the transition probability matrix in 2 months (4-step transition) can be written as $\mathbf{P}^{(4)} = \mathbf{P}^{4}$.

In order to investigate the effects of advice program and other smoking related factors, such as commence age of smoking, duration of smoking, and time of first cigarette in the morning on the transitions between states, we simultaneously applied three polytomous

logistic regression models to estimate the odds ratios of covariates for being in different states from “Precontemplation”, “Contemplation”, and “Preparation” with the following formulae,

$$\log\left(\frac{P_{ij}}{P_{ii}}\right) = \alpha_i + \beta_{ij}X \qquad i\text{=1, 2, 3, and ,}j\text{=1, 2, 3,4.}$$

, where X was the vector of covariates of interests mentioned above. Numbers of subjects in the four states followed multinomial distribution. The estimates and 95% credible intervals were obtained by applying the Bayesian Monte Carlo Markov Model simulation. Univariate polytomous logistic regression models with one covariate affecting all transition probabilities in separate models was applied, followed by the multi-variate polytomous logistic regression model including significant covariates in the univariate analyses.

The odds ratios (ORs) of covariates on the net force of forward transition from any given state *i* can be obtained by taking the exponential transform of the forward regression coefficients subtracted by the backward regression coefficients [2].

References

1. Cox DR and Miller HD. The theory of stochastic processes. London: Methuen, 1965.

2. Tseng CD, Yen AM, Chiu SYH, Chen LS, Chen HH, Chang SH. Predictive Model for Risk of Prehypertension and Hypertension and Expected Benefit after Population-based Life-style Modification (KCIS No. 24). Am J Hypertens. 2012;25(2):171-9. doi: 10.1038/ajh.2011.122

Supplementary Table S1. Univariate analysis of estimated odds ratio of transition departing from precontemplation, and Contemplation/Preparation

	**Precontemplation**		**Contemplation**		**Preparation**	
	OR	95% CI	OR	95% CI	OR	95% CI
Intervention group						
P&NA vs Control	0.34	0.02, 4.49	12.96*	1.49, 149.93	4.56	0.08, 152.78
Age						
per advanced year	0.30*	0.13, 0.71	0.27*	0.14, 0.53	2.25*	1.07, 4.67
Smoking commence age						
per advanced year	0.23*	0.12, 0.48	0.27*	0.14, 0.74	2.74*	1.10, 6.98
Duration of smoking						
per advanced year	0.32*	0.13, 0.73	0.31*	0.13, 0.79	3.36*	1.29, 7.47
First cigarette in the morning						
>=30 min vs <30 min	0.02*	0.00, 0.10	0.67	0.12, 4.02	1.81	0.27, 11.12
Cessation advice from others						
Yes vs No	0.01*	0.00, 0.05	1.24	0.21, 7.49	3.20	0.47, 17.64

* p<0.05

Supplementary Figure S1. Four-state Markov model for the stage changes of smoking cessation for male smokers

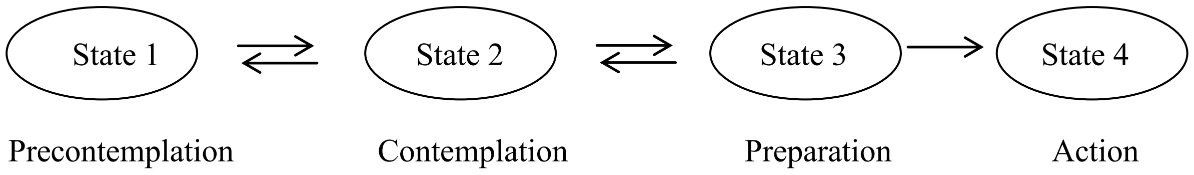

© 2020 Siewchaisakul P. et al.
